# Active surveillance of Methicillin-resistant *Staphylococcus aureus *in an emergency department: Comparing BD MAX StaphSR kit with the routine MRSA identification method

**DOI:** 10.1186/1757-7241-23-S1-A29

**Published:** 2015-07-16

**Authors:** Poul Kjældgaard, Christian B Morgensen, Lilli Ø Skov, Ming Chen

**Affiliations:** 1 Department of Clinical Microbiology, Southern Jutland Hospital, Aabenraa, Denmark; 2 Emergency Department, Southern Jutland Hospital, Aabenraa, Denmark

## Background

The area our hospital serves has a high prevalence of pig-MRSA and is situated close to Germany where there is a higher MRSA prevalence than in Denmark. The aim of the study was to describe the MRSA colonization among acutely admitted patients to the Emergency Department (ED) and to evaluate the BD MAX StaphSR screening kit versus our routine MRSA identification method.

## Methods

Prospective observational study performed at the ED. Nasal and throat swab specimens from all patients > 10 years who were admitted to the ED from 01.09.2013 to 30.11.2013, were examined. The swabs were, immediately after sampling, incubated in 6% NaCl broth for at least 16 hours and further cultivated on MRSA-chrome- and Columbia-agar. 150 ml of the incubated broth was used as sample material for StaphSR.

## Results

A total of 1,246 patients were included. In 11 patients (0.9%), MRSA was detected by our routine method, 5 isolates were pig-MRSA (CC398/T034), 4 Northern Germany MRSA in two different stains (CC22/t223 and CC5/t002), both having induced several MRSA-outbreaks in hospitals in Southern Denmark in last years. The last 2 strain were not formerly identified (CC45/t015 and CC88/t5147). 10 of the MRSA isolates were identified by the StaphSR kit, sensitivity 91% (95% CI: 62-98%) and specificity 98% (97-99%). 26 of the StaphSR results were false positive, mostly caused by presence of non-MRSA *S. aureus*, in 4 cases *S. epidermidis *, and in 7 cases a combination of non-MRSA *S. aureus *and *S. epidermidis*, resulting in a positive predictive value of the StaphSR kit of 28% (16-44%) StaphSR seemed to be sensitive to the amount of inoculum used. The 26 false positive results were reduced to 6 when adjusting the inoculum to 50 ml.

## Conclusions

The prevalence of MRSA in our ED was 0.9%, mainly with Pig-MRSA and Northern Germany MRSAstrains. StaphSR detected most of the culture positive samples, but gave nearly three times as many false positive results. All positive results should be confirmed and StaphSR kit cannot be recommended as the only method for MRSA detection.

